# Mesoscopic Simulation of the (2 + 1)-Dimensional Wave Equation with Nonlinear Damping and Source Terms Using the Lattice Boltzmann BGK Model

**DOI:** 10.3390/e21040390

**Published:** 2019-04-11

**Authors:** Demei Li, Huilin Lai, Baochang Shi

**Affiliations:** 1 College of Mathematics and Informatics, FJKLMAA, Fujian Normal University, Fuzhou 350117, China; 2 School of Mathematics and Statistics, Huazhong University of Science and Technology, Wuhan 430074, China

**Keywords:** lattice Boltzmann BGK model, Chapman-Enskog expansion, nonlinear damping, wave equation, hyperbolic telegraph equation, sine-Gordon equation

## Abstract

In this work, we develop a mesoscopic lattice Boltzmann Bhatnagar-Gross-Krook (BGK) model to solve (2 + 1)-dimensional wave equation with the nonlinear damping and source terms. Through the Chapman-Enskog multiscale expansion, the macroscopic governing evolution equation can be obtained accurately by choosing appropriate local equilibrium distribution functions. We validate the present mesoscopic model by some related issues where the exact solution is known. It turned out that the numerical solution is in very good agreement with exact one, which shows that the present mesoscopic model is pretty valid, and can be used to solve more similar nonlinear wave equations with nonlinear damping and source terms, and predict and enrich the internal mechanism of nonlinearity and complexity in nonlinear dynamic phenomenon.

## 1. Introduction

Nonlinear dynamic phenomenon, which exists in many fields of science and engineering, such as hydrodynamic, nonlinear optics, biology, plasma physics, and so on, can be modeled by many systems of nonlinear partial differential equations (NPDEs) [1,2]. The dynamical processes of these nonlinear systems are very important for both production and scientific research, and they should be studied by a suitable method designed to treat the nonlinear problems. Many researchers use different analytical or numerical methods to investigate various nonlinear dynamic systems. Because of the complexity and particularity of the nonlinear evolution equations, there is no unity approach to find every solution to the nonlinear dynamic systems. Consequently, how to construct accurate and available methods to solve the nonlinear evolution equations has been an absorbing research career. In recent decades, with the vigorous development of computer science and technology, researchers have developed many different types of numerical methods to obtain numerical solutions, including the finite element, finite difference, finite volume, variational iteration, and spectral methods, etc. [3].

In this work, the generalized (2 + 1)-dimensional dynamical equation with nonlinear damping and source terms is considered to be as follows:

(1) $$\frac{\partial^{2}u}{\partial t^{2}}+\alpha \frac{\partial u}{\partial t}=\beta \Delta u+f\left(x,y,t,u\right),\;\left(x,y\right)\in \Omega ,\;t\in \left[t_{0},T\right],$$

where Ω={(x,y):a≤x≤b,c≤y≤d}. The initial conditions associated with Equation (1) are given as follows:

(2) $$\begin{array}{c} \left\{\begin{array}{c} u\left(x,y,t_{0}\right)=\varphi_{1}\left(x,y\right),\;\left(x,y\right)\in \Omega \\ u_{t}\left(x,y,t_{0}\right)=\varphi_{2}\left(x,y\right),\;\left(x,y\right)\in \Omega , \end{array}\right. \end{array}$$

and the Dirichlet boundary conditions are given by

(3) $$\begin{array}{c} \left\{\begin{array}{c} u\left(a,y,t\right)=\phi_{1}\left(y,t\right),\;t\in \left[t_{0},T\right],\\ u\left(b,y,t\right)=\phi_{2}\left(y,t\right),\;t\in \left[t_{0},T\right],\\ u\left(x,c,t\right)=\phi_{3}\left(x,t\right),\;t\in \left[t_{0},T\right],\\ u\left(x,d,t\right)=\phi_{4}\left(x,t\right),\;t\in \left[t_{0},T\right], \end{array}\right. \end{array}$$

where u(x,y,t) is the scalar variable; *t* is the time; Δ is the Laplace operator; the parameter α,β are supposed to be real number with α,β≥0. α is the alleged dissipative term. When α=0, Equation (1) is degraded into the undamped wave equation, while α>0, to the damped one. The known functions $\varphi_{1}\left(x,y\right)$ and $\varphi_{2}\left(x,y\right)$ represent wave kinks or modes and velocity, respectively. and $\phi_{i},\left(i=1-4\right)$ are known functions of their arguments. In recent years, many scholars used different types of methods to obtain the numerical solution, such as the implicit Lie-group iterative scheme [4], the meshless method [5], the space-time spectral method [6], the compact finite difference method [7], the nonconforming quadrilateral finite element method [8].

Recently, the mesoscopic lattice Boltzmann method (LBM) has made significant progress in the research nonlinear dynamical equations and evolving process of complexity micro-mesoscopic systems [9], especially in fluid mechanics [10,11,12]. Unlike traditional macroscopic numerical methods, which are independent of the discrete macroscopic evolution equations, the LBM is based on the mesoscopic kinetic evolution equations with distribution functions when the expression of the equilibrium distribution function is known. The fundamental idea is to take the place of the differential evolution equations of nonlinear system by the discrete kinetic Boltzmann equations. To obtain the macroscopic fluid behavior, we just need to calculate the discrete Boltzmann equations to obtain the evolution of the distribution function. From a computational resource perspective, the remarkable merits are brevity of programming, numerical potency, inherent parallelism, and ease treatment of intricate boundary conditions. This kind of method has comprehensive capacities in quite several fields, from phonon transport [13] to approximate incompressible flows [14,15,16,17,18,19,20,21,22,23,24,25], full compressible flows [26,27,28,29,30,31,32,33,34,35,36,37], dendrite growth [38,39] and thermal multiphase flows [40]. Recently, the mesoscopic kinetics method is also becoming increasingly popular in computational mathematics and engineering science for solving certain NPDEs, including Burgers’ equations [41,42], Korteweg-de Vries equation [43], Gross-Pitaevskii equation [44], convection-diffusion equation [45,46,47,48,49,50,51], Kuramoto-Sivashinsky equation [52], wave equation [53,54], Dirac equation [55], Poisson equation [56] etc.

Inspired by the successful promotion and application of the mesoscopic LBM in modeling nonlinear convection-diffusion system [45,46], the aim of this work is to further develop and apply the lattice Boltzmann Bhatnagar-Gross-Krook (BGK) method to solve (2 + 1)-dimensional wave equation with nonlinear damping and source terms. In the process of linking the mesoscopic Boltzmann equation to the nonlinear damped evolution system, we should choose suitable local equilibrium distribution functions to meet some constraints.

The content of this work is as follows: the next section presents the mesoscopic Boltzmann BGK model and deduces the wave equation with nonlinear damping and source terms through the multiscale expansion technique. Numerical verification of the model is presented in Section 3. Finally, a summary of the research is given in the last section.

## 2. Lattice Boltzmann BGK Model

In the present lattice Boltzmann BGK model, we use a single relaxation factor model for collision terms in this work. The discrete Boltzmann equation of the model with the BGK model takes the form [45]

(4) $$\begin{array}{c} f_{j}\left(x+c_{j}\Delta t,t+\Delta t\right)=f_{j}\left(x,t\right)-\frac{1}{\tau }\left[f_{j}\left(x,t\right)-f_{j}^{eq}\left(x,t\right)\right]+\Delta tF_{j}\left(x,t\right)+\frac{\Delta t^{2}}{2}\frac{\partial F_{j}\left(x,t\right)}{\partial t}, \end{array}$$

where τ is the dimensionless relaxation time which regulates the rate of access to equilibrium state, $f_{j}\left(x,t\right)$ and $f_{j}^{eq}\left(x,t\right)$ are the distribution function and local equilibrium distribution function, respectively, and $F_{j}\left(x,t\right)$ is the distribution function for the source term. $\left\{c_{j},j=0,1,\cdots ,8\right\}$ is the collection of discrete directions of the particle velocity, for D2Q9 model, $\left\{c_{j},j=0,1,\cdots ,8\right\}=\left\{\left(0,0\right),\left(\pm c,0\right),\left(0,\pm c\right),\left(\pm c,\pm c\right)\right\}$, c=Δx/Δt, Δx is the spatial step, Δt is the time step.

In contrast to the common Lattice BGK model, we define the first derivative of u(x,t) as the following conservation condition [53]

(5) $$\frac{\partial u}{\partial t}=\sum_{j}f_{j}\left(x,t\right)=\sum_{j}f_{j}^{eq}\left(x,t\right).$$

To obtain the corresponding macroscopic evolution equation exactly, we should take $f_{j}^{eq}$ as

(6) $$\begin{array}{c} f_{j}^{eq}=w_{j}\left[\frac{\partial u}{\partial t}+\frac{c_{s}^{2}\left(u-\frac{\partial u}{\partial t}\right)I:\left(c_{j}c_{j}-c_{s}^{2}I\right)}{2c_{s}^{4}}\right], \end{array}$$

where the item I is the unit tensor, the item $c_{s}$ is referring to the sound speed, satisfy $c_{s}^{2}=c^{2}/3$. $w_{j}$ are the weights coefficients and satisfy the following conditions: $\sum_{j}w_{j}=1$, $\sum_{j}w_{j}c_{j}=0$, $\sum_{j}w_{j}c_{j}c_{j}=c_{s}^{2}I$, then $w_{0}=4/9,w_{1\;4}=1/9,w_{5,8}=1/36$.

Meanwhile, the corresponding source term $F_{j}$ is taken as

(7) $$\begin{array}{c} F_{j}=w_{j}F, \end{array}$$

where

(8) $$\begin{array}{c} F=f\left(x,t,u\right)-\alpha \frac{\partial u}{\partial t}. \end{array}$$

Then, $f_{j}^{eq}$ and $F_{j}$ should satisfy the following conservation conditions:

(9) $$\begin{array}{c} \left\{\begin{array}{c} \sum_{j}c_{j}f_{j}^{eq}=0,\\ \sum_{j}c_{j}c_{j}f_{j}^{eq}=c_{s}^{2}uI,\\ \sum_{j}F_{j}=F,\\ \sum_{j}c_{j}F_{j}=0, \end{array}\right. \end{array}$$

To obtain the macroscopic evolution Equation (1), we apply the Chapman-Enskog multiscale expansion to the distribution function, the first order time derivative, the spatial derivative and the source term as follows:

(10) $$\begin{array}{c} \left\{\begin{array}{c} f_{j}=f_{j}^{eq}+\varepsilon f_{j}^{\left(1\right)}+\varepsilon^{2}f_{j}^{\left(2\right)},\\ \frac{\partial }{\partial t}=\varepsilon \frac{\partial }{\partial t_{1}}+\varepsilon^{2}\frac{\partial }{\partial t_{2}},\\ \nabla =\varepsilon \nabla_{1},\\ F=\varepsilon F^{\left(1\right)}, \end{array}\right. \end{array}$$

where the item ε is as a small Knudsen number. Then, from Equation (7), and according to Equation (8), we obtain

(11) $$\begin{array}{c} \sum_{j}f_{j}^{\left(k\right)}=0\;\left(k\geq 1\right),\;\sum_{j}F_{j}^{\left(1\right)}=F^{\left(1\right)},\;\sum_{j}c_{j}F_{j}^{\left(1\right)}=0, \end{array}$$

where $F_{j}^{\left(1\right)}=w_{j}F^{\left(1\right)}$.

Employing the Taylor formula to discrete Boltzmann Equation (2) at point (x,t), we have

(12) $$\begin{array}{c} D_{j}f_{j}+\frac{\Delta t}{2}D_{j}^{2}f_{j}+\cdots =-\frac{1}{\tau \Delta t}\left(f_{j}-f_{j}^{eq}\right)+F_{j}+\frac{\Delta t}{2}\frac{\partial F_{j}}{\partial t}, \end{array}$$

where $D_{j}=\frac{\partial }{\partial t}+c_{j}\cdot \nabla$. Substitute Equation (10) into Equation (12), we have

(13) $$\begin{array}{c} \begin{array}{c} \left(\varepsilon D_{1j}+\varepsilon^{2}\frac{\partial }{\partial t_{2}}\right)\left(f_{j}^{eq}+\varepsilon f_{j}^{\left(1\right)}\right)+\frac{\Delta t}{2}{\left(\varepsilon D_{1j}+\varepsilon^{2}\frac{\partial }{\partial t_{2}}\right)}^{2}f_{j}^{eq}+\cdots \\ =-\frac{1}{\tau \Delta t}\left(\varepsilon f_{j}^{\left(1\right)}+\varepsilon^{2}f_{j}^{\left(2\right)}\right)+\varepsilon F_{j}^{\left(1\right)}+\varepsilon^{2}\frac{\Delta t}{2}\frac{\partial F_{j}^{\left(1\right)}}{\partial t_{1}}, \end{array} \end{array}$$

where $D_{1j}=\frac{\partial }{\partial t_{1}}+c_{j}\cdot \nabla_{1}$.

Then we derive the first- and second-order equation in ε as

(14) $$\begin{array}{c} O\left(\varepsilon^{1}\right):D_{1j}f_{j}^{eq}=-\frac{1}{\tau \Delta t}f_{j}^{\left(1\right)}+F_{j}^{\left(1\right)}, \end{array}$$

(15) $$\begin{array}{c} O\left(\varepsilon^{2}\right):\frac{\partial f_{j}^{eq}}{\partial t_{2}}+D_{1j}f_{j}^{\left(1\right)}+\frac{\Delta t}{2}D_{1j}^{2}f_{j}^{eq}=-\frac{1}{\tau \Delta t}f_{j}^{\left(2\right)}+\frac{\Delta t}{2}\frac{\partial F_{j}^{\left(1\right)}}{\partial t_{1}}. \end{array}$$

Multiplying both sides of Equation (14) by the operator $D_{1j}$, we obtain

(16) $$\begin{array}{c} D_{1j}^{2}f_{j}^{eq}=-\frac{1}{\tau \Delta t}D_{1j}f_{j}^{\left(1\right)}+D_{1j}F_{j}^{\left(1\right)}, \end{array}$$

then substitute Equation (16) into Equation (15), we get

(17) $$\begin{array}{c} \frac{\partial f_{j}^{eq}}{\partial t_{2}}+\left(1-\frac{1}{2\tau }\right)D_{1j}f_{j}^{\left(1\right)}+\frac{\Delta t}{2}c_{j}\cdot \nabla_{1}F_{j}^{\left(1\right)}=-\frac{1}{\tau \Delta t}f_{j}^{\left(2\right)}. \end{array}$$

Summing Equations (14) and (17) over *i* and using Equations (5), (9) and (11), we get

(18) $$\begin{array}{c} \frac{\partial^{2}u}{\partial t_{1}\partial t}=F^{\left(1\right)}, \end{array}$$

(19) $$\begin{array}{c} \frac{\partial^{2}u}{\partial t_{2}\partial t}+\left(1-\frac{1}{2\tau }\right)\nabla_{1}\cdot \sum_{j}c_{j}f_{j}^{\left(1\right)}=0. \end{array}$$

Based on Equation (14), and using Equations (9) and (11), we have

(20) $$\begin{array}{c} \begin{array}{ccc} \sum_{j}c_{j}f_{j}^{\left(1\right)} & = & -\tau \Delta t\sum_{j}c_{j}\left(D_{1j}f_{j}^{eq}-F_{j}^{\left(1\right)}\right)\\ & = & -\tau \Delta t\left(\frac{\partial }{\partial t_{1}}\sum_{j}c_{j}f_{j}^{eq}+\nabla_{1}\cdot \sum_{j}c_{j}c_{j}f_{j}^{eq}-\sum_{j}c_{j}F_{j}^{\left(1\right)}\right)\\ & = & -\tau \Delta tc_{s}^{2}\nabla_{1}u. \end{array} \end{array}$$

Then, substituting Equation (20) into Equation (19), we obtain

(21) $$\begin{array}{c} \frac{\partial^{2}u}{\partial t_{2}\partial t}-\tau \Delta tc_{s}^{2}\left(1-\frac{1}{2\tau }\right)\nabla_{1}\cdot \nabla_{1}u=0. \end{array}$$

Therefore, when Equation (18) ×ε + Equation (21) $\times \varepsilon^{2}$ is applied, we have

(22) $$\begin{array}{c} \frac{\partial^{2}u}{\partial t^{2}}-\tau \Delta tc_{s}^{2}\left(1-\frac{1}{2\tau }\right)\Delta u=F. \end{array}$$

To recover Equation (1) to order $O(\varepsilon^{2})$, we only need to let

(23) $$\begin{array}{c} \left\{\begin{array}{c} \beta =\tau \Delta tc_{s}^{2}\left(1-\frac{1}{2\tau }\right),\\ F=f\left(x,y,t,u\right)-\alpha \frac{\partial u}{\partial t}. \end{array}\right. \end{array}$$

so, we have

(24) $$\begin{array}{c} \tau =\frac{1}{2}+\frac{\beta }{\Delta tc_{s}^{2}}. \end{array}$$

In the calculation process, to get u(x,t), using Equation (5), and applying the difference scheme to the item $\frac{\partial u(x,t)}{\partial t}$, we have

(25) $$\sum_{j}f_{j}\left(x,t\right)=\frac{\partial u(x,t)}{\partial t}=\frac{u(x,t)-u(x,t-\Delta t)}{\Delta t},$$

then we get

(26) $$u\left(x,t\right)=\Delta t\sum_{j}f_{j}\left(x,t\right)+u\left(x,t-\Delta t\right).$$

## 3. Numerical Simulation

In this section, to show the efficiency of the present Lattice BGK model, we give some relevant numerical examples with and without damping terms. In addition, in order to compare with the exact solutions, the efficiency of present model is been tested. We set up the initial condition of distribution function $f_{j}\left(x,t\right)$ by setting to equal $f_{j}^{eq}\left(x,t\right)$ for all grid points at $t=t_{0}$. In addition, the macroscopic quantity u(x,t) in Equation (1) is also initialized by the given initial condition. The traditional explicit difference scheme $\partial_{t}F_{j}\left(x,t\right)=\left[F_{j}\left(x,t\right)-F_{j}\left(x,t-\Delta t\right)\right]/\Delta t$ can be used for calculating $\partial_{t}F_{j}\left(x,t\right)$, here we use the analytic expression. The non-equilibrium extrapolation of distribution function proposed by Guo et al. [57] is applied to handle the boundary conditions. The instructions for the detailed process of boundary treatment are basic and detailed below.

Notice that the distribution function $f_{j}$ can be decomposed into its equilibrium and non-equilibrium parts

(27) $$\begin{array}{c} f_{j}\left(x,t\right)=f_{j}^{\left(0\right)}\left(x,t\right)+f_{j}^{\left(neq\right)}\left(x,t\right), \end{array}$$

where $f_{j}^{\left(0\right)}$ and $f_{j}^{\left(neq\right)}$ are the equilibrium and the non-equilibrium parts of $f_{j}$.

Through the Chapman-Enskog multiscale analysis for the LBM, we can assume that $f_{j}^{\left(neq\right)}=\varepsilon f_{j}^{\left(1\right)}$. For better presentation, we assume $x_{b}$ is a boundary node, and $x_{f}$ is the nearest neighboring grid point of $x_{b}$ at a distance $c_{j}\Delta t$. Thus, the non-equilibrium part of the distribution at grid point $x_{f}$ can be given by

(28) $$\begin{array}{c} \varepsilon f_{j}^{\left(1\right)}\left(x_{f},t\right)=f_{j}^{\left(neq\right)}\left(x_{f},t\right)=f_{j}\left(x_{f},t\right)-f_{j}^{\left(0\right)}\left(x_{f},t\right). \end{array}$$

Notice that the grid point $x_{f}$ is the nearest neighboring grid point $x_{b}$ at a distance $\Delta x=\varepsilon c_{j}$, then we have $f_{i}^{\left(1\right)}\left(x_{b},t\right)=f_{i}^{\left(1\right)}\left(x_{f},t\right)+O\left(\varepsilon \right)$, and we obtain

(29) $$\begin{array}{c} \begin{array}{ccc} f_{j}\left(x_{b},t\right) & = & f_{j}^{\left(0\right)}\left(x_{b},t\right)+\varepsilon f_{j}^{\left(1\right)}\left(x_{b},t\right)\\ & = & f_{j}^{\left(0\right)}\left(x_{b},t\right)+\varepsilon \left[f_{i}^{\left(1\right)}\left(x_{f},t\right)+O\left(\varepsilon \right)\right]\\ & = & f_{j}^{\left(0\right)}\left(x_{b},t\right)+\varepsilon f_{i}^{\left(1\right)}\left(x_{f},t\right)+O\left(\varepsilon^{2}\right)\\ & = & f_{j}^{\left(0\right)}\left(x_{b},t\right)+f_{j}\left(x_{f},t\right)-f_{j}^{\left(0\right)}\left(x_{f},t\right)+O\left(\varepsilon^{2}\right), \end{array} \end{array}$$

where $f_{j}\left(x_{f},t\right)$ can be obtained by Equation (4), and $f_{j}^{\left(0\right)}\left(x_{b},t\right)$ and $f_{j}^{\left(0\right)}\left(x_{f},t\right)$ can be got by Equation (6). Therefore, as long as the macroscopic quantity of the boundary is given, the equilibrium distribution function of the boundary can be obtained, and the distribution function of the boundary can be obtained according to the above non-equilibrium extrapolation Formula (29).

Before simulation and calculation, we need to determine the expression of $F_{j}$ and $\partial_{t}F_{j}$ according to the source term *F* of the given macroscopic Equation (1), then we can obtain the concrete discrete expression (4).

Next, we will introduce the calculation procedures of the present model as follows:

**Step 1**: Based on the given conditions u(x,0) and $u_{t}\left(x,0\right)$ of Equation (2), initialize $f_{j}^{eq}\left(x,0\right)$ by Equation (6).

**Step 2**: Initialize $f_{j}\left(x,0\right)$ by $f_{j}^{eq}\left(x,0\right)$ from **Step 1** in all grid points.

**Step 3**: Calculate $f_{j}\left(x,t\right)$ of the inner points by the discrete Boltzmann Equation (4).

**Step 4**: Calculate u(x,t) by Equation (26) and $u_{t}\left(x,t\right)$ by Equation (5) of the inner points. If the specified termination time is reached, the program stops.

**Step 5**: Calculate u(x,t) and $u_{t}\left(x,t\right)$ of the boundary points by the given conditions (2).

**Step 6**: Calculate $f_{j}^{eq}\left(x,t\right)$ of the boundary points by Equation (6).

**Step 7**: Calculate $f_{j}\left(x,t\right)$ of the boundary points by Equation (29).

**Step 8**: Calculate $f_{j}\left(x,t+\Delta t\right)$ of all grid points by Equation (4), then return to **Step 4**.

With the present mesoscopic model, we simulate several known exact solutions of the second-order (2 + 1)-dimensional hyperbolic telegraph equation and the (2 + 1)-dimensional damped, driven sine-Gordon equation, respectively. Furthermore, the (2 + 1)-dimensional undamped sine-Gordon equation with different initial condition of various ring solitons are studied to understand the nonlinear behavior characteristics of the system.

Meanwhile, we adopt four different kinds of error norms for measuring the present model’s precision. The root mean square error norm $L_{2}$, max error norm $L_{\infty }$, global relative error norm GRE and root mean square error norm RMS are generally defined as

(1) The relative error norm ($L_{2}$-error)
  (30) $$L_{2}={\left(\sum_{i=1}^{N_{x}}\sum_{j=1}^{N_{y}}|u\left(x_{i},y_{j},t\right)-u^{*}\left(x_{i},y_{j},t\right){|}^{2}\right)}^{1/2}/{\left(\sum_{i=1}^{N_{x}}\sum_{j=1}^{N_{y}}|u^{*}\left(x_{i},y_{j},t\right){|}^{2}\right)}^{1/2}.$$
(2) The max error norm ($L_{\infty }$-error)
  (31) $$L_{\infty }=\max_{i,j}|u\left(x_{i},y_{j},t\right)-u^{*}\left(x_{i},y_{j},t\right)|.$$
(3) The global relative error norm (GRE-error)
  (32) $$\begin{array}{c} \mathrm{GRE}=\sum_{i=1}^{N_{x}}\sum_{j=1}^{N_{y}}|u\left(x_{i},y_{j},t\right)-u^{*}\left(x_{i},y_{j},t\right)|/\sum_{i=1}^{N_{x}}\sum_{j=1}^{N_{y}}\left|u^{*}\left(x_{i},y_{j},t\right)\right|. \end{array}$$
(4) The root mean square error norm (RMS-error)
  (33) $$\mathrm{RMS}={\left(\sum_{i=1}^{N_{x}}\sum_{j=1}^{N_{y}}|u\left(x_{i},y_{j},t\right)-u^{*}\left(x_{i},y_{j},t\right){|}^{2}/\left(N_{x}\times N_{y}\right)\right)}^{1/2}.$$

Here, $u(x_{i},y_{j},t)$, $u^{*}\left(x_{i},y_{j},t\right)$ are numerical solution and exact solution, respectively. The summation is added up from the information of all mesh points. Results show that the numerical solutions agree fairly well with the exact solutions over a considerable period of time.

**Example** **1.** Consider the following (2 + 1)-dimensional hyperbolic telegraph equation in the region 0≤x≤2, 0≤y≤2 as follows:

(34) $$\begin{array}{c} \begin{array}{c} \frac{\partial^{2}u}{\partial t^{2}}+2\frac{\partial u}{\partial t}+u=\frac{\partial^{2}u}{\partial x^{2}}+\frac{\partial^{2}u}{\partial y^{2}}-2e^{x+y-t}, \end{array} \end{array}$$

the initial conditions are given below

(35) $$\begin{array}{c} \left\{\begin{array}{c} u\left(x,y,0\right)=e^{x+y},\\ \frac{\partial u}{\partial t}\left(x,y,0\right)=-e^{x+y}. \end{array}\right. \end{array}$$

The exact solution for the current problem is given in Ref. [5] by

(36) $$\begin{array}{c} u\left(x,y,t\right)=e^{x+y-t}. \end{array}$$

The boundary conditions are given from the exact solution.

**Figure 1:**
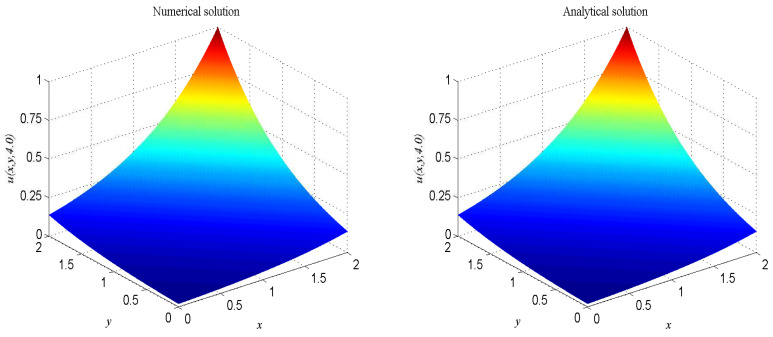
Spatio-temporal evolution of the numerical (**left**) and exact (**right**) solutions at t=4.0 for Example 1.

**Figure 2:**
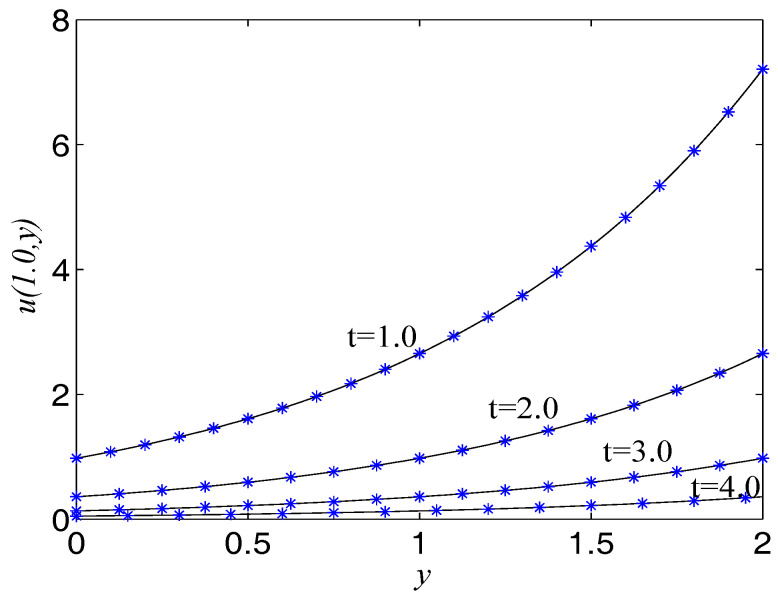
Comparison of numerical solutions with exact ones at x=1.0 at different times for Example 1. The solid lines are drawn as the exact solutions.

In numerical simulation, we take $F\left(x,t\right)=-u-2\partial u/\partial t-2e^{x+y-t}$, α=2, β=1, Δx=Δy=0.025, c=200. The computational domain is pinned to Ω=[0,2]×[0,2]. We present the surface graph of the numerical and exact solutions by the present lattice BGK model at t=4.0, see Figure 1. For clarity of contrast, we also demonstrate the two-dimensional contrast diagrams at x=1.0 for specific different times: t=1.0, t=2.0, t=3.0 and t=4.0, see Figure 2. The relative error norm $L_{2}$, the max error norm $L_{\infty }$, the global relative error GRE norm and the root mean square error RMS norm for the solutions of the second-order hyperbolic telegraph equation at different instants of time can be found in Table 1.

**Figure 3:**
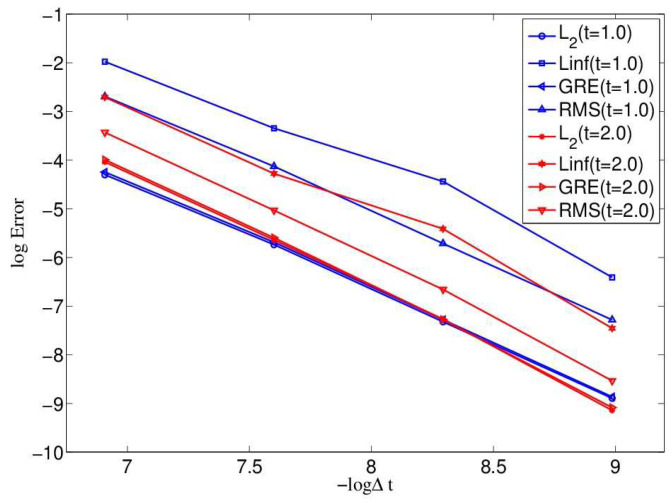
Accuracy test at t=1.0 and 2.0 with $N_{x}=N_{y}=80$ for Example 1.

To measure the accuracy of the present mesoscopic model, the relative error, max error, global relative error and root mean square error are shown in Figure 3 at different time t=1.0 and t=2.0 with different resolutions, range from $\Delta t=1.25\times 10^{-4}$ to $\Delta t=10^{-3}$ and c=25 to 200, with $N_{x}=N_{y}=80$. It is found that the present mesoscopic model is of second-order time accuracy. The order of the maximum error norm increases from 2.0720 to 2.2868, the order of the global relative error norm increases from 2.0789 to 2.2968, and the order of the root mean square error norm increases from 2.0720 to 2.2865.

**Example** **2.** Consider the following (2 + 1)-dimensional hyperbolic equation in the area 0≤x≤2, 0≤y≤2 given by

(37) $$\begin{array}{c} \begin{array}{c} \frac{\partial^{2}u}{\partial t^{2}}+\frac{\partial u}{\partial t}=\frac{\partial^{2}u}{\partial x^{2}}+\frac{\partial^{2}u}{\partial y^{2}}-2\sin u+2\sin\left[e^{-t}\left(1-\cos\left(\pi x\right)\right)\left(1-\cos\left(\pi y\right)\right)\right]\\ -\pi^{2}e^{-t}\left[\cos\left(\pi x\right)+\cos\left(\pi y\right)-2\cos\left(\pi x\right)\cos\left(\pi y\right)\right], \end{array} \end{array}$$

the initial conditions are given below

(38) $$\begin{array}{c} \left\{\begin{array}{c} u(x,y,0)=(1-\cos(\pi x))(1-\cos(\pi y)),\\ \frac{\partial u}{\partial t}\left(x,y,0\right)=-\left(1-\cos\left(\pi x\right)\right)\left(1-\cos\left(\pi y\right)\right). \end{array}\right. \end{array}$$

The exact solution for this instance is given in Ref. [6] by

(39) $$\begin{array}{c} u\left(x,y,t\right)=e^{-t}\left(1-\cos\left(\pi x\right)\right)\left(1-\cos\left(\pi y\right)\right). \end{array}$$

The boundary conditions are given from the exact solution from Equation (39).

**Figure 4:**
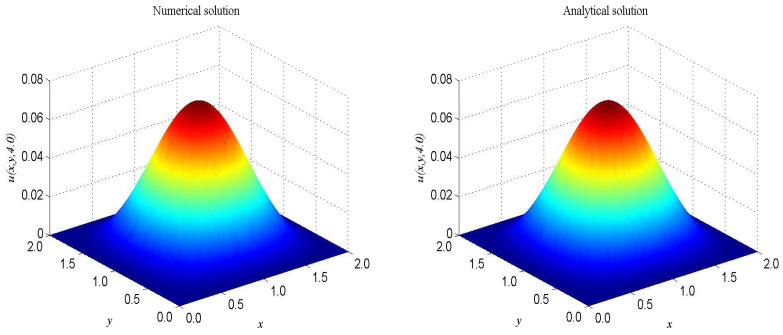
Spatio-temporal evolution of the numerical (**left**) and exact (**right**) solutions at t=4.0 for Example 2.

**Figure 5:**
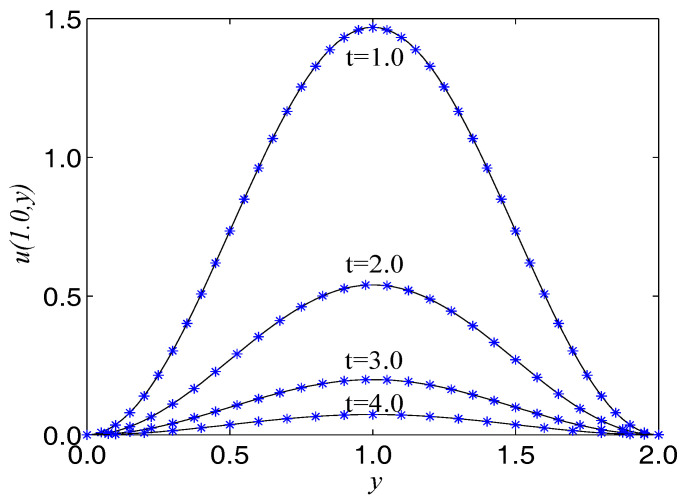
Comparison of numerical solutions with exact ones at x=1.0 at different instants of time for Example 2. The solid lines are drawn as the exact solutions.

The term ’sinu’ is a very good representation of nonlinearity. In the proceeding, we take $F\left(x,t\right)=-2\sin u+2\sin\left[e^{-t}\left(1-\cos\left(\pi x\right)\right)\left(1-\cos\left(\pi y\right)\right)\right]-\pi^{2}e^{-t}\left[\cos\left(\pi x\right)+\cos\left(\pi y\right)-2\cos\left(\pi x\right)\cos\left(\pi y\right)\right]$, α=β=1, Δx=Δy=0.025, c=200. The computational domain is pinned to Ω=[0,2]×[0,2]. We present the spatio-temporal evolution of the numerical and exact solutions by the present model at t=4.0, see Figure 4. For clarity of contrast, we also present the two-dimensional contrast diagrams at x=1.0 for especial different times: t=1.0, t=2.0, t=3.0 and t=4.0, see Figure 5. The maximum value of the wave decays slowly over time due to the damping term and the source term. The relative error $L_{2}$, max error norm $L_{\infty }$, global relative error norm GRE and root mean square error norm RMS for the solutions of the second-order hyperbolic telegraph equation at specific times can be found in Table 2.

**Example** **3.** Consider the following (2 + 1)-dimensional hyperbolic equation in the area 0≤x≤1, 0≤y≤1 given by

(40) $$\begin{array}{c} \begin{array}{c} \frac{\partial^{2}u}{\partial t^{2}}+4\pi \frac{\partial u}{\partial t}+2\pi^{2}u=\frac{\partial^{2}u}{\partial x^{2}}+\frac{\partial^{2}u}{\partial y^{2}}+2\pi t\sin\left[\pi \left(x+y\right)\right]e^{-(x+y)t}\\ +\left[{\left(x+y-2\pi \right)}^{2}-2t^{2}\right]\sin\left(\pi x\right)\sin\left(\pi y\right)e^{-(x+y)t}, \end{array} \end{array}$$

the initial conditions are given below

(41) $$\begin{array}{c} \left\{\begin{array}{c} u(x,y,0)=\sin(\pi x)\sin(\pi y),\\ \frac{\partial u}{\partial t}\left(x,y,0\right)=-\sin\left(\pi x\right)\sin\left(\pi y\right). \end{array}\right. \end{array}$$

The exact solution for this instance is given in Ref. [7] by

(42) $$\begin{array}{c} u\left(x,y,t\right)=e^{-(x+y)t}\sin\left(\pi x\right)\sin\left(\pi y\right). \end{array}$$

The boundary conditions are given from the exact solution.

**Figure 6:**
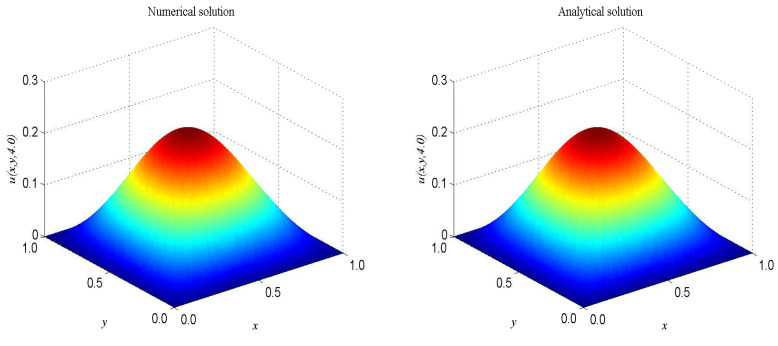
Spatio-temporal evolution of the numerical (**left**) and exact (**right**) solutions at t=4.0 for Example 3.

**Figure 7:**
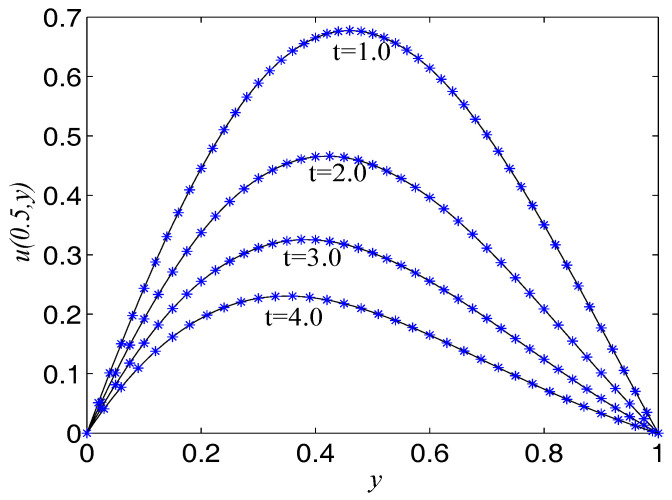
Comparison of numerical solutions with exact ones at x=0.5 at specific times for Example 3. The solid lines are drawn as the exact solutions.

In the proceeding, we take $F\left(x,t\right)=-u-2\left(1+\pi^{2}\right)\partial u/\partial t$, $\alpha =2(1+\pi^{2})$, β=1, Δx=Δy=0.0125, c=500. The computational domain is pinned to Ω=[0,1]×[0,1]. We present the spatio-temporal evolution of the numerical and exact solutions by the LBM at t=4.0, see Figure 6. For clarity of contrast, we also present the two-dimensional contrast diagrams at x=0.5 for some specific times: t=1.0, t=2.0, t=3.0 and t=4.0, see Figure 7. The relative error norm $L_{2}$, max error norm $L_{\infty }$, global relative error norm GRE and root mean square error norm RMS for the solutions of the second-order hyperbolic telegraph equation at specific times can be found in Table 3.

**Example** **4.** Consider the following (2 + 1)-dimensional hyperbolic equation in the area 0≤x≤1, 0≤y≤1 given by

(43) $$\begin{array}{c} \begin{array}{c} \frac{\partial^{2}u}{\partial t^{2}}+\frac{1}{2}\frac{\partial u}{\partial t}+4u=\frac{\partial^{2}u}{\partial x^{2}}+\frac{\partial^{2}u}{\partial y^{2}}+2\left(\pi^{2}+2\right)e^{-0.5t}\sin\left(\pi x\right)\sin\left(\pi y\right), \end{array} \end{array}$$

the initial conditions are given below

(44) $$\begin{array}{c} \left\{\begin{array}{c} u(x,y,0)=\sin(\pi x)\sin(\pi y),\\ \frac{\partial u}{\partial t}\left(x,y,0\right)=-0.5\sin\left(\pi x\right)\sin\left(\pi y\right). \end{array}\right. \end{array}$$

The exact solution for this instance is given in Ref. [7] by

(45) $$\begin{array}{c} u\left(x,y,t\right)=e^{-0.5t}\sin\left(\pi x\right)\sin\left(\pi y\right). \end{array}$$

The boundary conditions are given from the exact solution.

**Figure 8:**
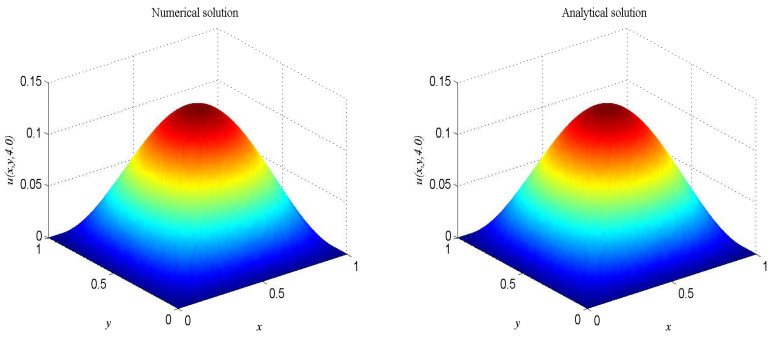
Spatio-temporal evolution of the numerical (**left**) and exact (**right**) solutions at t=4.0 for Example 4.

**Figure 9:**
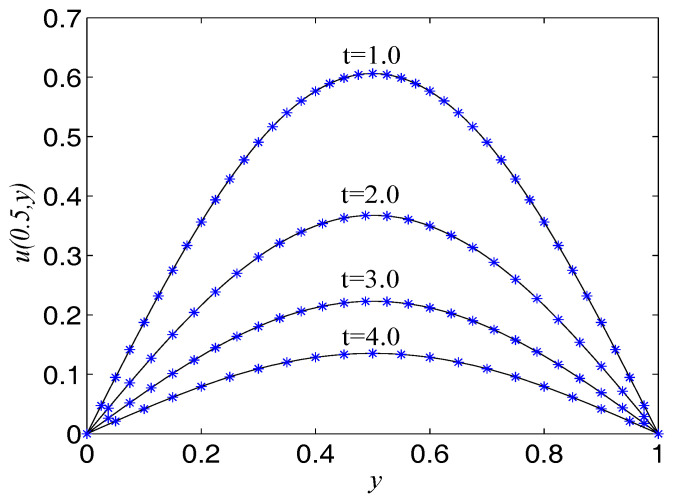
Comparison of numerical solutions with exact ones at x=0.5 at specific times for Example 4. The solid lines are drawn as the exact solutions.

In the proceeding, we take $F\left(x,t\right)=-4u-0.5\partial u/\partial t+2\left(\pi^{2}+2\right)e^{-0.5t}\sin\left(\pi x\right)\sin\left(\pi y\right)$, α=0.5, β=1, Δx=Δy=0.0125, c=500. The computational domain is pinned to Ω=[0,1]×[0,1]. We present the spatio-temporal evolution of the numerical and exact solutions by the present model at t=4.0, see Figure 8. For clarity of contrast, we also present the two-dimensional contrast diagrams at x=0.5 for specific different times: t=1.0, t=2.0, t=3.0 and t=4.0, see Figure 9. The relative error norm $L_{2}$, max error norm $L_{\infty }$, global relative error norm GRE and root mean square error norm RMS for the solutions of the second-order hyperbolic telegraph equation at specific times can be found in Table 4.

**Example** **5.** Consider the following (2 + 1)-dimensional sine-Gordon equation [58] given by

(46) $$\begin{array}{c} \begin{array}{c} \frac{\partial^{2}u}{\partial t^{2}}=\frac{\partial^{2}u}{\partial x^{2}}+\frac{\partial^{2}u}{\partial y^{2}}-\sin u, \end{array} \end{array}$$

in the area −a<x<a, −b<y<b.

We simulate some particular cases of specific initial conditions with various numbers of circular ring solutions to study the nonlinear behaviors of the system. Numerical examples are carried out for three cases:

The first initial condition of one ring solitons is as follows:

(47) $$\begin{array}{c} \left\{\begin{array}{c} u\left(x,y,0\right)=\alpha^{\prime }\arctan\left[\exp(3-\sqrt{x^{2}+y^{2}})\right],\\ \frac{\partial u}{\partial t}\left(x,y,0\right)=0, \end{array}\right. \end{array}$$

where −14≤x,y≤14.

The second initial condition of two ring solitons is the following:

(48) $$\begin{array}{c} \left\{\begin{array}{c} u\left(x,y,0\right)=\alpha^{\prime }\sum_{j=1}^{2}\arctan\exp\gamma^{\prime }\left[4-\sqrt{{\left(x+x_{j}\right)}^{2}+{\left(y+y_{j}\right)}^{2}}\right],\\ \frac{\partial u}{\partial t}\left(x,y,0\right)=\beta^{\prime }\sum_{j=1}^{2}\mathrm{sech}\gamma^{\prime }\left[4-\sqrt{{\left(x+x_{j}\right)}^{2}+{\left(y+y_{j}\right)}^{2}}\right], \end{array}\right. \end{array}$$

where −30≤x≤10, −21≤y≤7, $\left\{\left(x_{j},y_{j}\right)\right\}=\left\{\left(3,7\right),\left(17,7\right)\right\}$.

The third initial condition of four ring solitons is as follows:

(49) $$\begin{array}{c} \left\{\begin{array}{c} u\left(x,y,0\right)=\alpha^{\prime }\sum_{j=1}^{4}\arctan\exp\gamma^{\prime }\left[4-\sqrt{{\left(x+x_{j}\right)}^{2}+{\left(y+y_{j}\right)}^{2}}\right],\\ \frac{\partial u}{\partial t}\left(x,y,0\right)=\beta^{\prime }\sum_{j=1}^{4}\mathrm{sech}\gamma^{\prime }\left[4-\sqrt{{\left(x+x_{j}\right)}^{2}+{\left(y+y_{j}\right)}^{2}}\right], \end{array}\right. \end{array}$$

where −30≤x≤10, −30≤y≤10, $\left\{\left(x_{j},y_{j}\right)\right\}=\left\{\left(3,3\right),\left(3,17\right),\left(17,3\right),\left(17,17\right)\right\}$.

**Figure 10:**
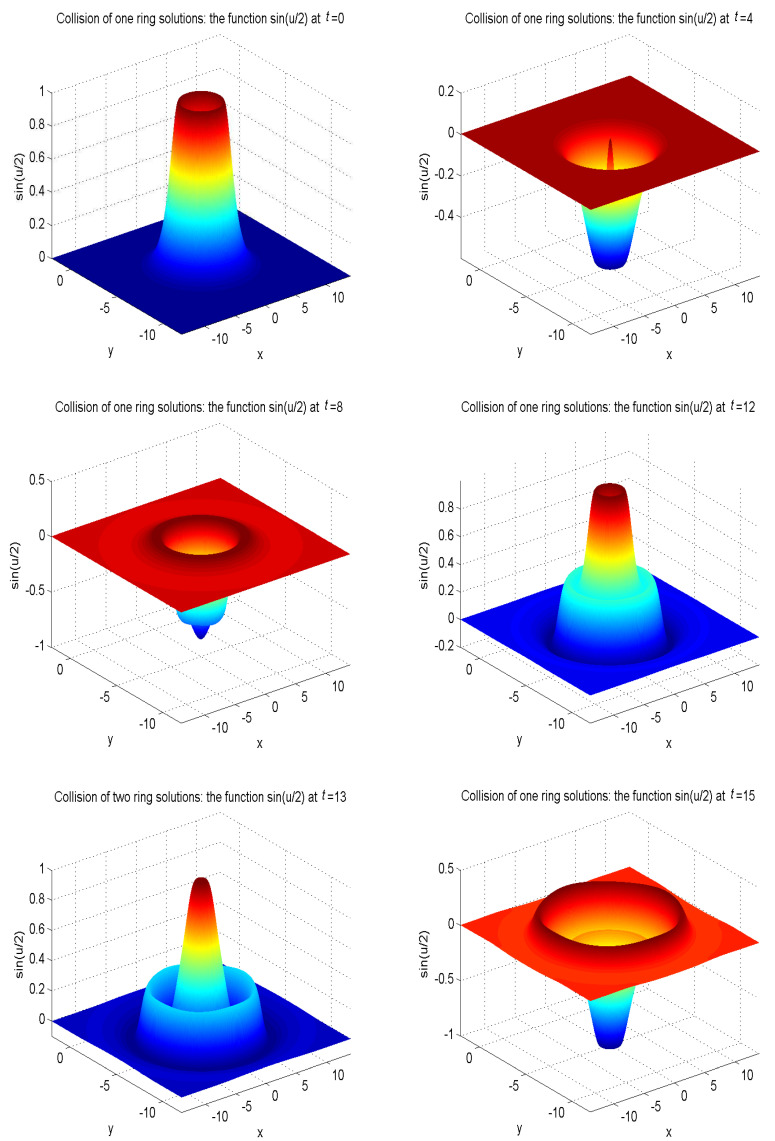
Collision of one ring solitons: the function sin(u/2) at t=0, t=4.0, t=8.0, t=12.0, t=13.0, t=15.0, for Example 5 with the initial condition (47).

**Figure 11:**
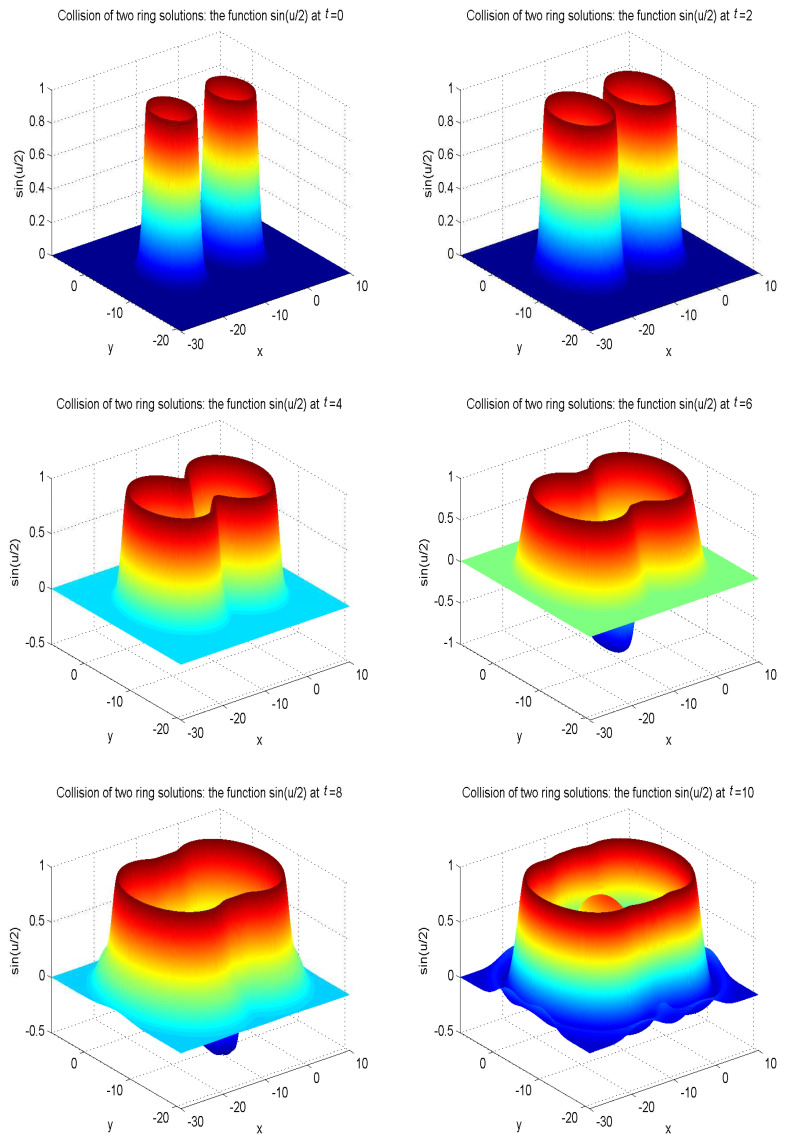
Collision of two ring solitons: the function sin(u/2) at t=0, t=2.0, t=4.0, t=6.0, t=8.0, t=10.0, for Example 5 with the initial condition (48).

**Figure 12:**
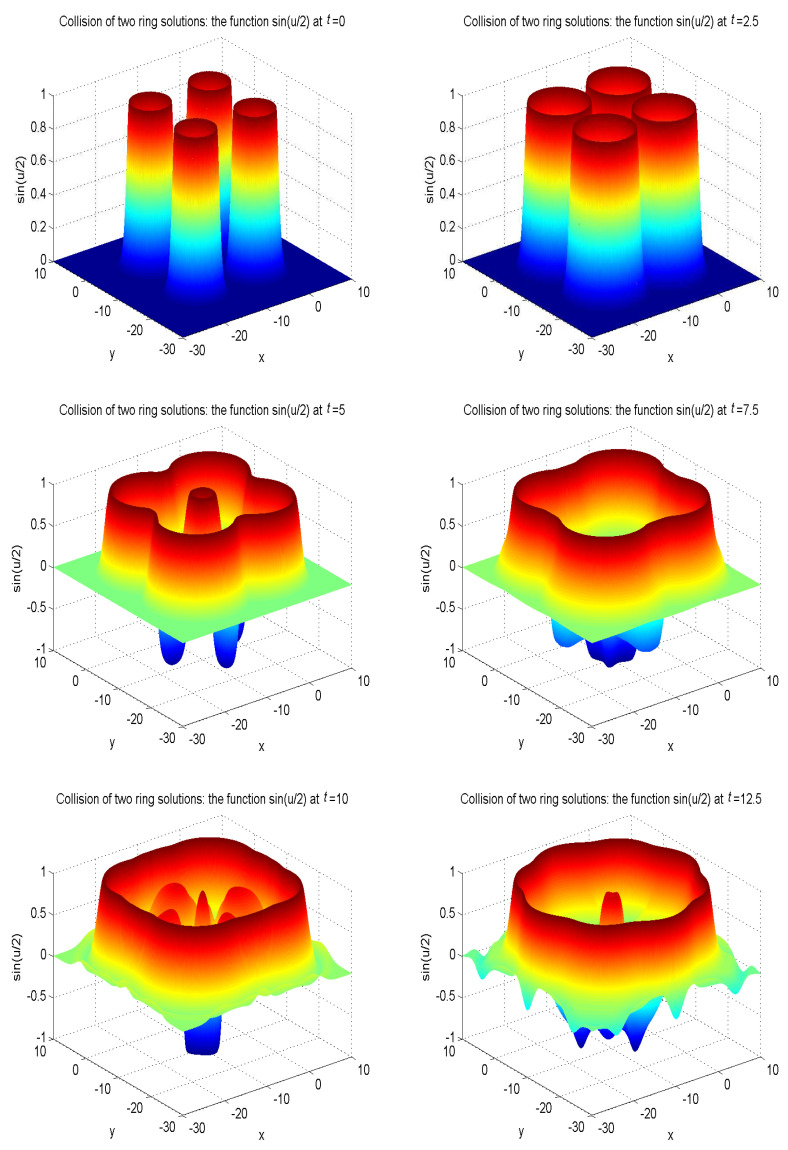
Collision of four ring solitons: the function sin(u/2) at t=0, t=2.5, t=5.0, t=7.5, t=10.0, t=12.5 with the initial condition (49).

In our simulations, the zero gradient is used to deal with the boundary of the domain as $u\left(x_{b},t\right)=u\left(x_{f},t\right)$, where $x_{b}$ is a boundary node, and $x_{f}$ is the nearest neighboring grid point of $x_{b}$ at a distance $c_{j}\Delta t$. The model parameters are set as $\alpha^{\prime }=4$, $\beta^{\prime }=4.13$, $\gamma^{\prime }=1/0.436$. The other parameters are given as α=1, β=0, Δx=Δy=0.05, c=50. To better display the nonlinear propagation process of the wave, we present the surface graph of numerical solutions of collision of one ring solitons in regard to sin(u/2) at t=0, t=4.0, t=8.0, t=12.0, t=13.0, t=15.0, see Figure 10. The surface graph of numerical solutions of collision of two ring solitons in regard to sin(u/2) at t=0, t=4.0, t=8.0, t=12.0, t=13.0, t=15.0, see Figure 11. The surface graph of numerical solutions of collision of four ring solitons in regard to sin(u/2) at t=0, t=2.5, t=5.0, t=7.5, t=10.0, t=12.5, see Figure 12.

It can be found that the solitons reveal potent nonlinear evolution characteristics as time passed. One of the distinct features of solitons is that they can evolve without phanic changes in their identity after interplay. These numerical simulation results are in accordance with those results in Ref. [58].

## 4. Conclusions

Based on the mesoscopic lattice BGK method, we have investigated the numerical solution of (2 + 1)-dimensional wave equation with nonlinear damping and source terms, such as the hyperbolic telegraph equation, damped or undamped sine-Gordon equation, and so on. With the help of the Chapman-Enskog multiscale expansion, the macroscopic dynamical evolution equation can be precisely obtained from the present mesoscopic scheme in the continuity system without appending any amending term. Through observation, we can find that for the sine-Gordon system without damping terms and other source terms, the crest of the wave will oscillate up and down, and at the same time, the waveform will deform in the form of two or four crests that have evolved into one over time. All these phenomena reflect the evolution characteristics of nonlinear systems. Numerical examples for some test issues have been held to check the present mesoscopic model. The numerical solutions are in well coincident with the exact ones. From the convergence research of the Example 1, it can be found that the present mesoscopic model has the second-order accuracy in time. It is believed that with this model, we can predict and enrich the characterization and description of the nonlinear behavior characteristics in complex nonlinear dynamic systems.

## Figures and Tables

**Table 1:** The maximum error $L_{2}$, relative error $L_{\infty }$ norm, global relative error norm GRE and root mean square error norm RMS for u(x,y) at different times in Example 1.

t	1.0	2.0	3.0	4.0
$L_{2}$	$1.3739\times 10^{-4}$	$1.0701\times 10^{-4}$	$3.9440\times 10^{-5}$	$1.5354\times 10^{-4}$
$L_{\infty }$	$1.6459\times 10^{-3}$	$5.7798\times 10^{-4}$	$2.1405\times 10^{-4}$	$8.2911\times 10^{-5}$
GRE	$1.4147\times 10^{-4}$	$1.1327\times 10^{-4}$	$3.4975\times 10^{-4}$	$1.6388\times 10^{-4}$
RMS	$6.8635\times 10^{-4}$	$1.9667\times 10^{-4}$	$2.6666\times 10^{-5}$	$3.8189\times 10^{-5}$

**Table 2:** The maximum error norm $L_{2}$, relative error norm $L_{\infty }$, global relative error norm GRE and root mean square error norm RMS for u(x,y) at specific instants of time in Example 2.

t	1.0	2.0	3.0	4.0
$L_{2}$	$6.6512\times 10^{-4}$	$1.1301\times 10^{-3}$	$1.7607\times 10^{-3}$	$2.3686\times 10^{-3}$
$L_{\infty }$	$1.2614\times 10^{-3}$	$4.5286\times 10^{-4}$	$4.2965\times 10^{-4}$	$1.3513\times 10^{-4}$
GRE	$8.3720\times 10^{-4}$	$1.4311\times 10^{-3}$	$2.0109\times 10^{-3}$	$2.7853\times 10^{-3}$
RMS	$3.6249\times 10^{-4}$	$2.2658\times 10^{-4}$	$1.2987\times 10^{-4}$	$6.4271\times 10^{-5}$

**Table 3:** The maximum error norm $L_{2}$, relative error norm $L_{\infty }$, global relative error norm GRE and root mean square error norm RMS for u(x,y) at specific times in Example 3.

t	1.0	2.0	3.0	4.0
$L_{2}$	$1.0885\times 10^{-4}$	$1.0395\times 10^{-4}$	$1.6103\times 10^{-4}$	$3.3706\times 10^{-4}$
$L_{\infty }$	$4.8807\times 10^{-5}$	$2.7630\times 10^{-5}$	$1.8712\times 10^{-5}$	$2.0280\times 10^{-5}$
GRE	$1.0063\times 10^{-4}$	$9.2696\times 10^{-5}$	$1.7823\times 10^{-4}$	$3.8410\times 10^{-4}$
RMS	$2.1102\times 10^{-5}$	$8.9647\times 10^{-6}$	$6.9148\times 10^{-6}$	$7.9351\times 10^{-6}$

**Table 4:** The maximum error norm $L_{2}$, relative error norm $L_{\infty }$, global relative error norm GRE and root mean square error norm RMS for u(x,y) at specific times in Example 4.

t	1.0	2.0	3.0	4.0
$L_{2}$	$9.7057\times 10^{-5}$	$3.4711\times 10^{-4}$	$2.8500\times 10^{-4}$	$1.5080\times 10^{-4}$
$L_{\infty }$	$5.9367\times 10^{-5}$	$1.2789\times 10^{-4}$	$6.3485\times 10^{-5}$	$2.0128\times 10^{-5}$
GRE	$9.6015\times 10^{-5}$	$3.4693\times 10^{-4}$	$2.8454\times 10^{-4}$	$1.5180\times 10^{-4}$
RMS	$2.9071\times 10^{-5}$	$6.3060\times 10^{-5}$	$3.1404\times 10^{-5}$	$1.0078\times 10^{-5}$
